# Impact of a patient fixation device on healthcare worker radiation doses in fluoroscopy-assisted endoscopy

**DOI:** 10.1093/rpd/ncaf103

**Published:** 2025-09-13

**Authors:** Masaki Onoe, Nobuhiko Fukuba, Yasuhide Kodama, Satoshi Kotani, Akihiko Oka, Naoki Oshima, Kotaro Shibagaki, Kousaku Kawashima, Norihisa Ishimura, Shunji Ishihara

**Affiliations:** Department of Gastroenterology and Hepatology, Shimane University School of Medicine, Izumo, Shimane, Japan; Department of Gastroenterology and Hepatology, Shimane University School of Medicine, Izumo, Shimane, Japan; Department of Gastroenterology and Hepatology, Shimane University School of Medicine, Izumo, Shimane, Japan; Department of Gastroenterology and Hepatology, Shimane University School of Medicine, Izumo, Shimane, Japan; Department of Gastroenterology and Hepatology, Shimane University School of Medicine, Izumo, Shimane, Japan; Department of Gastroenterology and Hepatology, Shimane University School of Medicine, Izumo, Shimane, Japan; Department of Endoscopy, Shimane University Hospital, Izumo, Shimane, Japan; Department of Gastroenterology and Hepatology, Shimane University School of Medicine, Izumo, Shimane, Japan; Department of Gastroenterology and Hepatology, Shimane University School of Medicine, Izumo, Shimane, Japan; Department of Gastroenterology and Hepatology, Shimane University School of Medicine, Izumo, Shimane, Japan

## Abstract

Background and aims: Healthcare professionals involved in fluoroscopy-guided endoscopy are occupationally exposed to ionizing radiation. We evaluated whether a patient-immobilization device, MEDO V-Fix®, reduces this exposure. Methods: Monthly effective and lens equivalent dose were measured for nurses and doctors using personal dosemeters worn inside protective gear. Data from 7 months before and 10 months after device introduction were compared. Additionally a cost–benefit analysis (CBA) of dose reduction was performed. Results: Monthly effective doses fell in both professions, but neither decline reached statistical significance. By contrast, nurses’ mean lens-equivalent dose dropped sharply from 35.0 to 6.5 μSv per procedure (*P* < 0.01), whereas the reduction in doctors was not significant. In the CBA, assuming a 5-y service life, the benefit-to-cost ratio ranged 1.02–2.72, indicating economic merit. Conclusions: The MEDO V-Fix significantly reduces the occupational radiation exposure of endoscopy nurses and is a worthwhile investment from a CBA perspective.

## Introduction

Interventional procedures such as endoscopic retrograde cholangiopancreatography (ERCP) and endoscopic ultrasonography (EUS) procedures have increased alongside the rising incidence of pancreatic and biliary cancers, especially in developed countries [1–4]. However, occupational exposure to ionizing radiation is a growing concern in healthcare, particularly among professionals involved in these procedures [5]. These procedures require real-time fluoroscopic imaging, which increases cumulative radiation doses to medical staff [4, 6].

Healthcare workers such as nurses and endoscopists are frequently positioned near radiation sources during these procedures, and existing protective measures like lead aprons and thyroid shields may not fully prevent exposure—especially to vulnerable areas such as the eyes. Accumulated exposure has been linked to long-term risks, including cataracts and other radiation-induced complications. Thus, enhancing workplace safety through practical, procedural interventions remains a critical priority [7, 8].

In this study, we evaluated the effect of the vacuum patient immobilization device MEDO V-Fix® on occupational radiation exposure. The main purpose of MEDO V-Fix is to support the safety of endoscopic procedures by suppressing sudden patient movements. As a result, it is expected that occupational radiation exposure will be reduced by reducing the number of personnel required to restrain the patient. In this study, we aimed to clarify the effect of MEDO V-Fix in reducing occupational radiation exposure.

## Materials and methods

**Figure 1:**
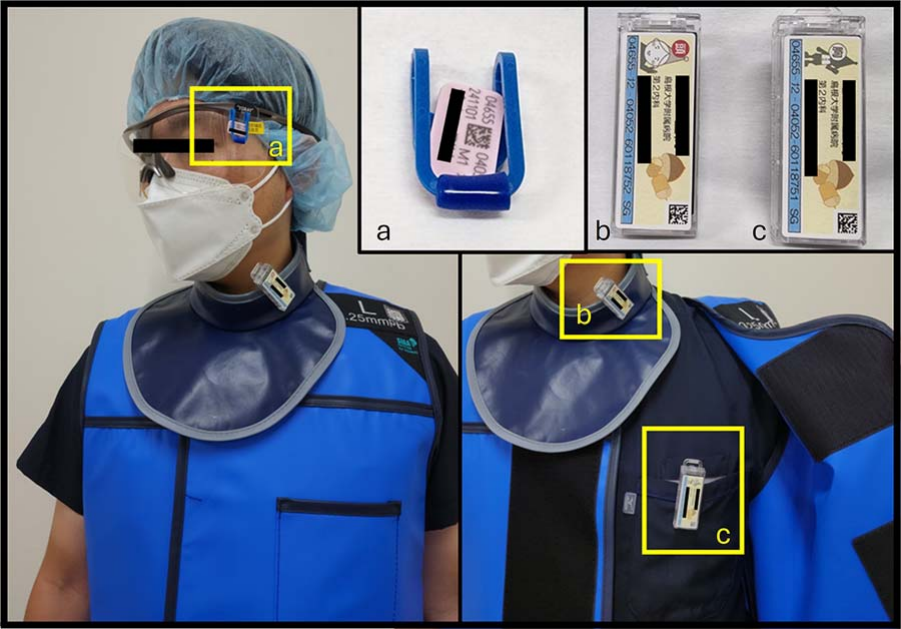
Optically stimulated luminescence Dosemeters. (a) Lens dose dosemeter for personnel with higher neck-area radiation exposure. (b) and (c) Effective doses measured with two dosemeters: One inside the lead protector (chest for men, abdomen for women) and one outside at neck level.

This single-center, retrospective observational study was conducted at Shimane University Hospital and approved by Medical Research Ethics Committee, Shimane University Faculty of Medicine (20230215–2). Radiation doses, including effective dose and lens equivalent dose, were measured monthly for doctors and nurses from the central radiology department over a period extending from April 2022 to August 2023. The effective doses were recorded using two optically stimulated luminescence dosemeters per staff member: one worn inside the lead protector (on the chest for men and the abdomen for women) and the other worn outside the protector in the neck area. According to the method published online [9] by the manufacturer of the dosemeters used in our hospital, the effective dose can be expressed as “(effective dose) = 0.11 × (head and neck) + 0.89 × (chest or abdomen)”. Personnel who regularly received higher radiation doses in the neck area also wore additional lens dosemeter inside their lead glasses (Fig. 1). In addition, staff members whose monthly lens-equivalent dose exceeded the departmental alert level were advised to implement additional radiation-protection measures so that their projected annual exposure would remain below the institutional annual dose limit (15 mSv).

The study recorded the monthly sum of individual radiation doses for each profession. Monthly occupational radiation dose was evaluated with two procedure-normalized indicators:

Doctors’ monthly total dose per procedure


$$ \frac{\sum{\mathrm{Dose}}_{\mathrm{doctors}}}{\mathrm{No}.\ \mathrm{of}\ \mathrm{procedures}\ \mathrm{in}\ \mathrm{the}\ \mathrm{month}}\left(\mathrm{\mu} \mathrm{Sv}/\mathrm{procedure}\right) $$

Nurses’ monthly total dose per procedure


$$ \frac{\sum{\mathrm{Dose}}_{\mathrm{nurses}}}{\mathrm{No}.\,\mathrm{of}\ \mathrm{procedures}\ \mathrm{in}\ \mathrm{the}\ \mathrm{month}}\left(\mathrm{\mu} \mathrm{Sv}/\mathrm{procedure}\right) $$

All doses were recorded by a personal luminescent dosemeter worn at chest or abdominal level inside a lead apron. Because the average monthly endoscopy volume was lower after the introduction of the MEDO V-Fix, normalizing by procedure count allowed fair comparison of exposure levels across the pre- and post-intervention periods. Radiation protection curtains were routinely used during all fluoroscopic procedures throughout the study period. This practice remained consistent before and after the introduction of the MEDO V-Fix, ensuring that any observed changes in radiation exposure were not influenced by variations in the use of other protective measures.

**Figure 2:**
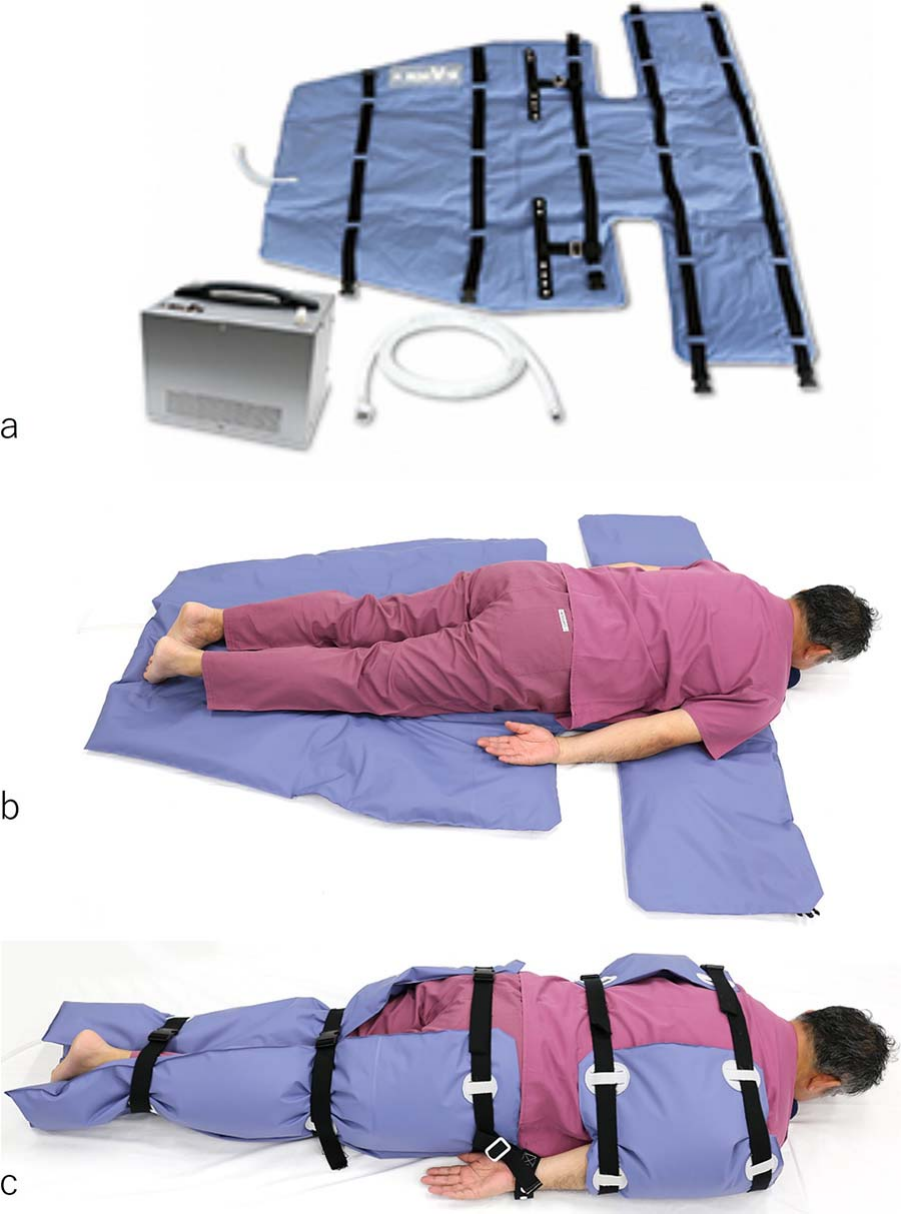
(a) The MEDO V-fix patient fixation device, comprising the main body, connecting hose, and mat. The main body contains the vacuum pressure generator, which connects to the mat via the hose. (b) The mat before wrapping the patient, showing its flat configuration. (c) The mat after wrapping the patient but prior to vacuum sealing, illustrating its positioning around the patient in preparation for immobilization.

The MEDO V-Fix was employed to secure patients in a prone position (Fig. 2). The device consists of a vacuum pressure generator that creates a vacuum in the mat, allowing the beads within the mat to adhere to each other and solidify according to the patient’s body shape, thus preventing unnecessary movement during the procedure. Sedation was administered before the mat was cured, ensuring patient comfort throughout the process. Sedation protocols, including the use of midazolam, pentazocine, and dexmedetomidine hydrochloride, were consistently applied before and after the introduction of the MEDO V-Fix, in accordance with institutional guidelines. This ensured that any differences in radiation exposure were not influenced by variations in sedation practices.

During the study period, patient immobilization devices, including MEDO V-fix, were not utilized in any fluoroscopic endoscopic procedures, including ERCP and EUS in the initial phase (April to October 2022). In this phase, assistant doctors and nurses manually immobilized the patient. In the subsequent phase (November 2022 to August 2023), the patient was immobilized with MEDO V-fix. This consistent principle of use ensures that the impact of the device is comprehensively evaluated across all relevant procedures where patient immobilization and radiation safety are critical.

For each variable, normality was assessed with the Shapiro–Wilk test. To compare the pre-intervention period (April–October 2022) with the post-intervention period (November 2022–August 2023), we proceeded as follows: when both groups conformed to a normal distribution, Welch’s *t*-test was applied and the *P*-value together with the 95% confidence interval (CI) were reported; when at least one group deviated from normality, the Mann–Whitney *U* test was used and only the *P*-value was reported. All analyses were performed with EZR software (version 1.54), and statistical significance was set at *P* < 0.05.

In addition, a cost–benefit analysis (CBA) was performed on the dose reduction effect of introducing MEDO V-Fix, referring to the report by Engström *et al.* [10, 11]. The annual collective dose was calculated from the individual effective dose to the chest or abdomen. The annual dose reduction before and after the introduction of MEDO V-Fix was calculated and multiplied by the service life of MEDO V-Fix to calculate the dose reduction for the service life. The monetary benefit of occupational dose reduction (B) was estimated as the product of the recommended monetary value per unit dose (α, US dollars /man−mSv), the annual collective dose reduction achieved by the intervention (ΔS, man-mSv/y), and the expected service life of the device in years (L).


$$ B=\alpha \times \Delta S\times L $$

The cost (C) of introducing MEDO V-Fix was calculated and evaluated using B/C. If B/C > 1, it was a socially useful investment, if B/C≒1, it was judged based on operability and safety considerations, and if B/C < 1, it was a too expensive investment.

## Results

The number of fluoroscopic endoscopies was 1251 (average 179 procedures/month) in the first half and 1601 (average 160 procedures/month) in the second half, with no significant difference.

**Figure 3:**
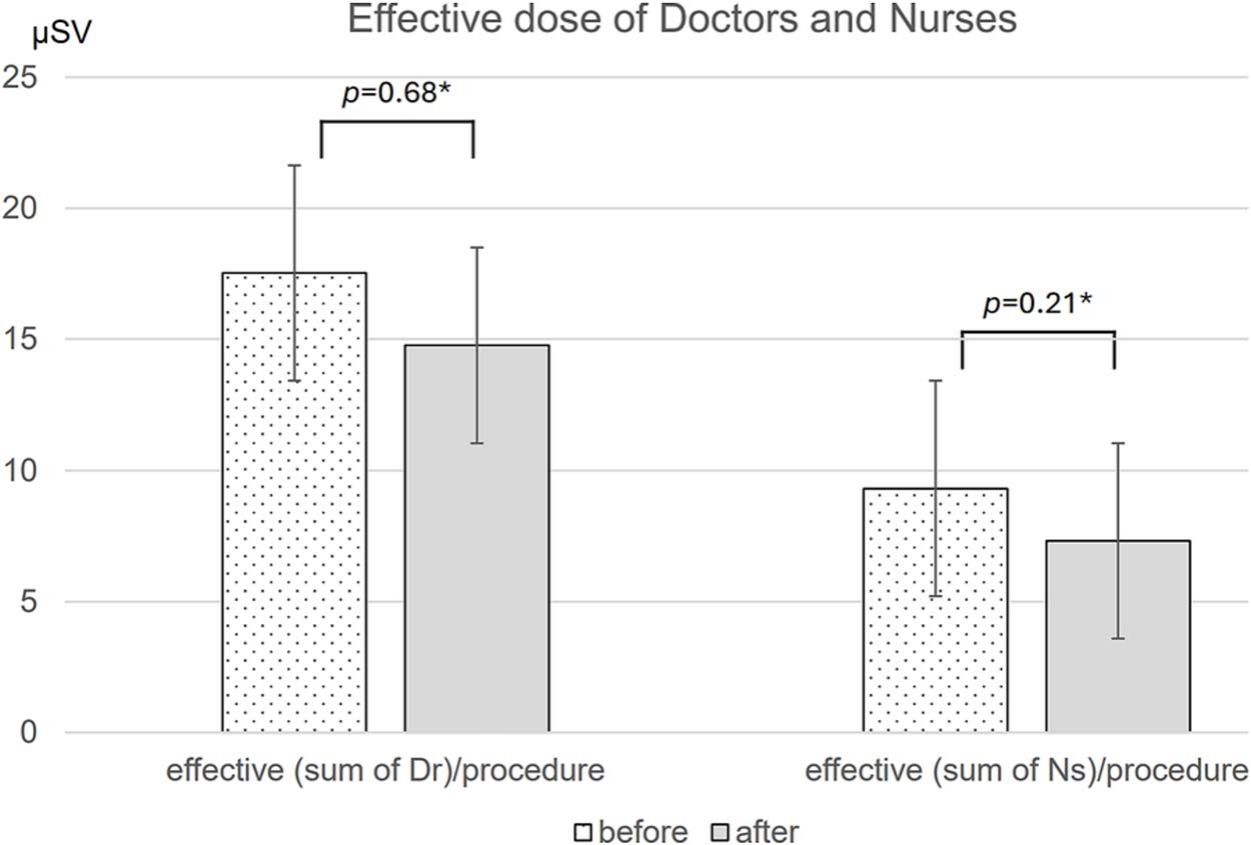
The monthly average of the total effective dose per case for doctors and nurses were shown (*: Welch T-test).

**Figure 4:**
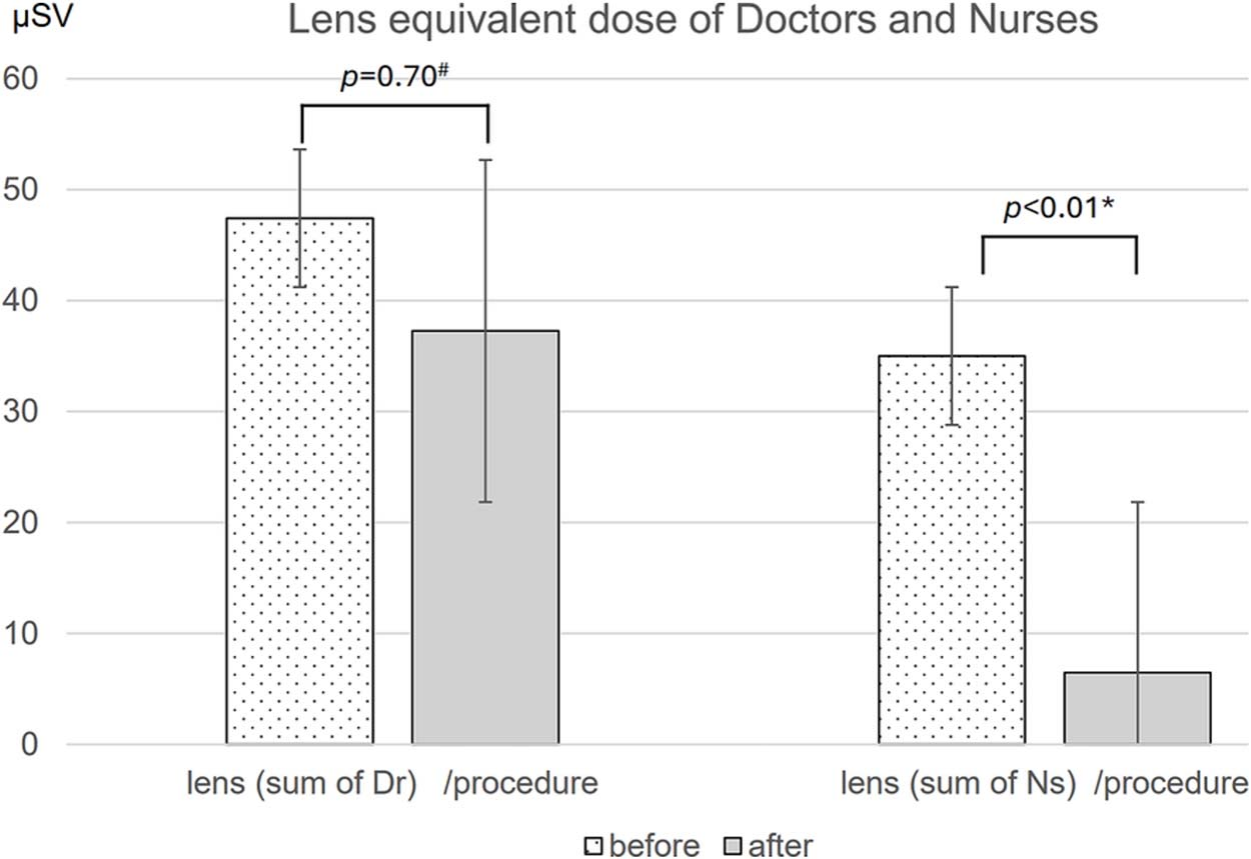
The monthly average of the total lens equivalent dose for doctors and nurses were shown (^#^: Mann–Whitney U-test,*: Welch T-test).

After the introduction of the MEDO V-Fix, a downward trend in effective doses normalized by endoscopic procedure count was observed among doctors and nurses (Fig. 3). The monthly average of the effective dose fell from 17.5 to 14.8 μSv per procedure for doctors (15% decrease; *P* = 0.68; 95% CI, −10.0 to 15.5 μSv) and from 9.3 to 7.3 μSv per procedure for nurses (22% decrease; *P* = 0.21; 95% CI, −1.1 to 5.1 μSv); neither reduction reached statistical significance. In contrast, the introduction of the MEDO V-Fix had an effect on the radiation exposure of the lens (Fig. 4). The monthly average of the total lens equivalent dose for nurses decreased significantly from 35.0 μSv per procedure before the introduction of the device to 6.5 μSv per procedure afterward (reduction by 81%, *P* = 0.003, 95%CI 16.4–40.7 μSv). Although there was no significant differences, the monthly average of the total lens equivalent dose for doctors tended to decrease from 47.4 μSv per procedure to 37.3 μSv per procedure (*P* = 0.70, 95%CI was not shown).

The CBA is shown in Table 1. The total individual effective dose for all occupations over a 6-month period before and after the introduction of MEDO V-Fix was 24.5 mSv and 13.1 mSv, respectively, with a 6-month dose reduction of 11.4 mSv and a 1-y effective dose reduction (ΔS) of 22.4 mSv (man-mSv/y). The service life of the MEDO V-Fix main body was 6 y, but considering that the hose and mat are consumables, the service life of the entire system (L) was set to be 5 y. From the above, the dose reduction for the 5-y service life of MEDO V-Fix was 114 mSv. However, since the service life depends on the careful handling, an error of ±2 y was assumed, and the dose reduction for 3 y and 7 y was 68.4 mSv and 159.6 mSv, respectively. The recommended monetary value (α) for Japanese workers was multiplied by 61–162 US dollars (USD)/man-mSv for the year 2023, and the minimum and maximum of the dose reduction monetary value (B) of MEDO V-Fix were 6954–18 468 USD for a 5-y service life. Even if the useful life was assumed to be 3 y, the cost was 4172.4–11080.8 USD, and if it was assumed to be 7 y, the cost was 9735.6–25 855.2 USD. On the other hand, the cost (C) of introducing MEDO V-Fix was 980,000 yen at the manufacturer’s suggested retail price, which was 6920 USD based on the exchange rate in 2023. A CBA was calculated using B/C. For a standard useful life of 5 y, B/C = 1.02–2.72, and it was evaluated as a socially desirable investment [10, 11]. Furthermore, if the useful life was estimated to be 7 y, B/C = 1.43–3.80. On the other hand, when the service life was estimated as 3 y and α was estimated as the most conservative, B/C was 0.61, which was evaluated as a too expensive investment.

**Table 1:** A CBA of MEDO V-fix.

			α(USD/man-mSv)	
			α_min_ = 61	α_max_ = 162
Estimated service life (L)	3 y (−2 y)	B(USD)	4172.2	11 080.8
		B/C	0.61	1.63
	5 y (standard)	B(USD)	6954	18 468
		B/C	1.02	2.72
	7 y (−2 y)	B(USD)	9735.6	25 855.2
		B/C	1.43	3.80

The total effective dose reduction for 1 y (ΔS) was 22.4 mSv (man-mSv/y). The cost (C) of introducing MEDO V-Fix was 980 000 yen at the manufacturer’s suggested retail price, which was 6920 USD based on the exchange rate in 2023.

## Discussion

The results of this study clearly demonstrate that the introduction of the MEDO V-Fix patient fixation device reduced occupational radiation exposure, especially for nurses involved in biliopancreatic endoscopy procedures. While the reduction in exposure among doctors was not statistically significant, the overall trend was consistent with the intended effects of radiation protection measures. These findings align with previous studies, such as those reported by Biegala *et al.* [12], which emphasized the importance of optimizing radiological protection during interventional radiology procedures to prevent excessive occupational exposure. Their study, focusing on vascular radiology procedures, highlighted that if the principles of radiological protection are not properly implemented, the permissible dose limits for body, eye lenses, and hands may be exceeded. In this study, the MEDO V-Fix reduced the need for manual patient restraint during endoscopic procedures, which in turn reduced the close contact between nurses and patients, a known source of radiation exposure. This result is consistent with the findings of Nessipkhan *et al.* [13], who reported significant improvements in radiation protection management practices following the introduction of new regulations and protective devices in Japanese hospitals. These improvements include better compliance with the use of dosemeters and protective equipment, which may have contributed to the overall reduction in radiation doses observed in this study. Furthermore, the reduction in exposure doses, particularly in the lens area, is notable, as occupational radiation exposure to the eyes has been linked to long-term health risks such as cataracts. Radiation-induced cataracts are a deterministic effect, with experimental and epidemiological data indicating a threshold dose of roughly 500 mSv; above this level, the probability of lens opacities rises sharply [14]. Consistent with these concerns, Parikh *et al.* [15] demonstrated that novel radiation protection devices significantly reduced head-level radiation exposure among catheterization laboratory personnel. These findings reinforce the importance of utilizing advanced radiation protection measures like the MEDO V-Fix to minimize radiation-related health risks among healthcare workers.

The results of radiation exposure reduction effects differed between doctors and nurses in this study. Nurses, who are often required to perform tasks in close proximity to the patient during endoscopic procedures, were likely to have experienced a greater reduction in radiation exposure following the introduction of the MEDO V-Fix. This result is consistent with studies such as that by Oishi *et al.* [16], which highlighted the higher levels of anxiety among radiology nurses due to their perceived vulnerability to radiation exposure. By reducing the need for close physical contact with the patient, the MEDO V-Fix effectively alleviated this occupational risk, leading to lower overall radiation exposure for nurses. In contrast, doctors primarily focus on the operation of endoscopic devices and the monitoring of fluoroscopic images, which likely resulted in them benefiting less from the radiation reduction effects of the MEDO V-Fix. As a result, the impact of the MEDO V-Fix on reducing radiation exposure for doctors was less pronounced. This difference in exposure levels between doctors and nurses highlights the varying risk profiles among healthcare workers involved in radiological procedures, as previously discussed by Seo *et al.* [17], who identified occupational variations in radiation exposure based on job roles and proximity to the radiation source.

The lens equivalent dose of nurses was significantly reduced after the introduction of MEDO V-Fix, but regarding the effective dose, there was no significant reduction. This is because the effective dose was calculated from values measured inside a lead apron, while the lens equivalent dose was measured inside lead glasses. The lead apron reduced the dose by up to 90%, while the lead glasses reduced the dose by up to ⁓50% [18, 19], and it is possible that these differences in protective wear influenced the effectiveness of MEDO-V Fix. The fact that MEDO-V Fix had a dose reduction effect on the total individual doses suggests that it may have a dose reduction effect that is not limited to certain staff members. This observation is consistent with the findings of Lisko *et al.* [20], who reported that radiation protection devices such as Rampart significantly reduced total-body radiation exposure for all members of the catheter insertion team, with no significant individual differences.

The present study also highlights the need for continued improvements in radiation protection practices. In addition to the MEDO V-Fix, other protective measures, including the use of lead aprons, thyroid shields, and protective curtains, played a role in reducing occupational radiation exposure. As reported by Biegała *et al.* [12], the use of modern radiological equipment and adherence to radiological protection principles are essential for minimizing radiation exposure during complex interventional procedures. Future studies should explore the potential benefits of integrating additional protective devices, such as the Protego system evaluated by Parikh *et al.* [15], which showed promise in further reducing radiation doses for all members of the procedural team. In the context of occupational health, such interventions can contribute not only to immediate dose reduction but also to the long-term prevention of radiation-related health issues, such as cataracts and potential carcinogenic effects. Further implementation across diverse procedural environments may reinforce a culture of safety and promote adherence to occupational exposure limits.

The cost–benefit ratios (B/C) performed in this study ranged from 1.02 to 2.72, indicating that under standard service life assumptions, the device is a socially desirable investment. Engström *et al.* [10] also emphasized that CBA is most informative when both costs and collective dose can be monetized, while qualitative benefits should be evaluated separately. In the present context, additional non-monetized advantages—such as shorter procedure times and reduced musculoskeletal strain on staff—would further improve the overall benefit of MEDO V-Fix, suggesting that the above B/C values are conservative. Taken together, these findings indicate that MEDO V-Fix is a cost-effective ALARA [21] implementation tool for Japanese endoscopy suites, especially when a service life of 5 y or longer can be secured. Ongoing post-implementation monitoring of measured doses and actual costs, combined with dynamic CBA that accommodates future changes in α values and service-life variability, will enhance the transparency and robustness of future decision-making.

This study has several limitations. First, Dose Area Product (DAP)—a key metric of patient radiation burden during fluoroscopy-guided endoscopy such as ERCP and, by extension, a major determinant of staff exposure—was not recorded; future prospective work that captures DAP will be needed to clarify the true impact of MEDO V-Fix. Second, because the study is retrospective, we could not audit badge compliance or positioning, leaving room for measurement error. Third, although radiation dose was corrected for the number of endoscopic procedures performed before and after MEDO V-Fix introduction, detailed fluoroscopy-time data were unavailable, limiting adjustment for procedural complexity. Finally, during ERCP the primary operator and assistant frequently switch roles and change their orientation to the X-ray source, so we could not examine left- versus right-sided dosemeter placement or compare doses between surgeons and assistants.

## Conclusion

The MEDO V-Fix effectively reduces occupational radiation exposure, particularly for nurses. Our CBA further shows that its adoption is economically justifiable as well as radiologically advantageous. Continued optimization of radiation-protection practices, together with the introduction of innovative protective devices, is essential to safeguard the long-term health of healthcare professionals involved in radiological procedures.

## Data Availability

Data generated or analyzed during this study are available from the corresponding author on reasonable request.

## Approval of the research protocol by an Institutional Reviewer Board

This study was reviewed and approved by the Ethics Committee of Shimane University (Approval Number: 20230215–2).
